# DNA Damage Response Gene Signature as Potential Treatment Markers for Oral Squamous Cell Carcinoma

**DOI:** 10.3390/ijms24032673

**Published:** 2023-01-31

**Authors:** Silvia Pomella, Matteo Cassandri, Ombretta Melaiu, Francesco Marampon, Marco Gargari, Vincenzo Campanella, Rossella Rota, Giovanni Barillari

**Affiliations:** 1 Department of Clinical Sciences and Translational Medicine, University of Rome Tor Vergata, Via Montpellier, 00133 Rome, Italy; 2 Department of Oncohematology, Bambino Gesù Children’s Hospital, IRCCS, 00146 Rome, Italy; 3 Department of Radiological Sciences, Oncology and Anatomical Pathology, Sapienza University of Rome, 00161 Rome, Italy

**Keywords:** oral squamous cell carcinoma, DNA damage response, The Cancer Genome Atlas, DepMap, bioinformatics

## Abstract

Oral squamous cell carcinoma (OSCC) is a rapidly progressive cancer that often develops resistance against DNA damage inducers, such as radiotherapy and chemotherapy, which are still the standard of care regimens for this tumor. Thus, the identification of biomarkers capable of monitoring the clinical progression of OSCC and its responsiveness to therapy is strongly required. To meet this need, here we have employed Whole Genome Sequencing and RNA-seq data from a cohort of 316 patients retrieved from the TCGA Pan-Cancer Atlas to analyze the genomic and transcriptomic status of the DNA damage response (DDR) genes in OSCC. Then, we correlated the transcriptomic data with the clinical parameters of each patient. Finally, we relied on transcriptomic and drug sensitivity data from the CTRP v2 portal, performing Pearson’s correlation analysis to identify putative vulnerabilities of OSCC cell lines correlated with DDR gene expression. Our results indicate that several DDR genes show a high frequency of genomic and transcriptomic alterations and that the expression of some of them correlates with OSCC grading and infection by the human papilloma virus. In addition, we have identified a signature of eight DDR genes (namely *CCNB1*, *CCNB2*, *CDK2*, *CDK4*, *CHECK1*, *E2F1*, *FANCD2*, and *PRKDC*) that could be predictive for OSCC response to the novel antitumor compounds sorafenib and tipifarnib-P1. Altogether, our data demonstrate that alterations in DDR genes could have an impact on the biology of OSCC. Moreover, here we propose a DDR gene signature whose expression could be predictive of OSCC responsiveness to therapy.

## 1. Introduction

Oral squamous cell carcinoma (OSCC) is one of the most common neoplasms of the head and neck region, accounting for over 90% of those developing in the oral cavity [1]. It originates from the malignant transformation of epithelial cells lining the oral cavity, with special regard to the palate, the floor of the mouth, and the tongue [1]. The global annual number of new OSCC cases is growing, with the highest peak in Asia followed by Western countries, placing OSCC among the ten most prevailing types of human cancer [2].

OSCC onset is influenced by factors that are constitutive of the patient (age, genotype) or related to his/her lifestyle (e.g., smoking, alcohol consumption, ultraviolet radiation, and/or the use of betel quid) [3]. As a matter of fact, OSCC is endemic in Asian countries, where about 50% of the cases are connected to betel quid chewing [4]. Human papillomavirus (HPV) infection has been linked to oropharyngeal carcinoma and some types of OSCC [5]. Worthy of note is the frequent detection of HPV in the OSCCs of the base of the tongue in patients with no previous exposure to other risk factors for OSCC [6].

OSCC treatment options are based on the severity of the disease and include surgery, radiation therapy, chemotherapy, or a combination of these modalities [7]. However, these therapeutic regimens have major side effects which negatively impact the quality of life of OSCC patients [8]. Moreover, patients which initially respond to the treatment may on time develop resistance which renders OSCC prognosis poor [9]. Furthermore, promising strategies such as neoadjuvant immunotherapy can be applied only to a small percentage of OSCC patients [10]. For these reasons, the survival of OSCC patients has remained approximately unchanged in the last few decades [11]. Therefore, the identification of molecular biomarkers orienting the selection of patients for personalized therapies is urgently needed.

In this regard, one should consider that the antitumor effect of chemo- or radiotherapy depends on their capability of damaging the DNA of cancer cells [12].

Actually, throughout life, human DNA is repeatedly damaged by exogenous (ultraviolet light, ionizing radiation, chemicals, toxins) or endogenous (reactive oxygen species, lipid peroxidation) agents. Opportunely, in the vast majority of cases, DNA lesions are repaired through activation of the DNA damage response (DDR) pathway [13,14]. The latter is a complex network of genes among which are the non-homologous end joining (NHEJ) and the homologous recombination (HR) [12]. DDR members detect DNA lesions and thereafter halt the cell cycle to allow DNA damage repair; in case this would not be possible, they induce programmed cell death [13,14].

Of importance, mutations and/or alterations in the DDR genes leading to their functional impairment are often found in cancer cells [15,16]. The combination of reduced DNA repair ability and increased DNA damage levels in cancer cells gives rise to further DNA mutations or chromosomal aberrations, resulting in genomic instability [17,18]. The latter, in turn, drives tumor initiation and progression by promoting oncogene activation and/or tumor suppressor loss [15]. In addition, genomic instability increases cellular heterogeneity within the tumor, thereby augmenting the chance of selecting radio- and/or chemo-resistant cells which eventually cause tumor relapses [16].

However, it is widely accepted that defects in the DDR genes may represent valuable prognostic/diagnostic markers and therapeutic targets for tumors [15,16].

Based on this evidence, in the current study, we have evaluated the alteration status and the expression of the DDR genes in a cohort of OSCC patients retrieved from The Cancer Genomic Atlas (TCGA) database. We have employed bioinformatic analyses to identify dysregulated genes, thus uncovering a correlation between the expression of (i) ten DDR genes and OSCC grading; (ii) thirteen DDR genes and HPV infection; and (iii) an eight-gene-based signature that could predict OSCC responsiveness to sorafenib and tipifarnib-P1, two drugs employed to treat this neoplasm [19,20].

## 2. Results

### 2.1. DDR Gene Alterations and Expressions in OSCC Patients

**Figure 1:**
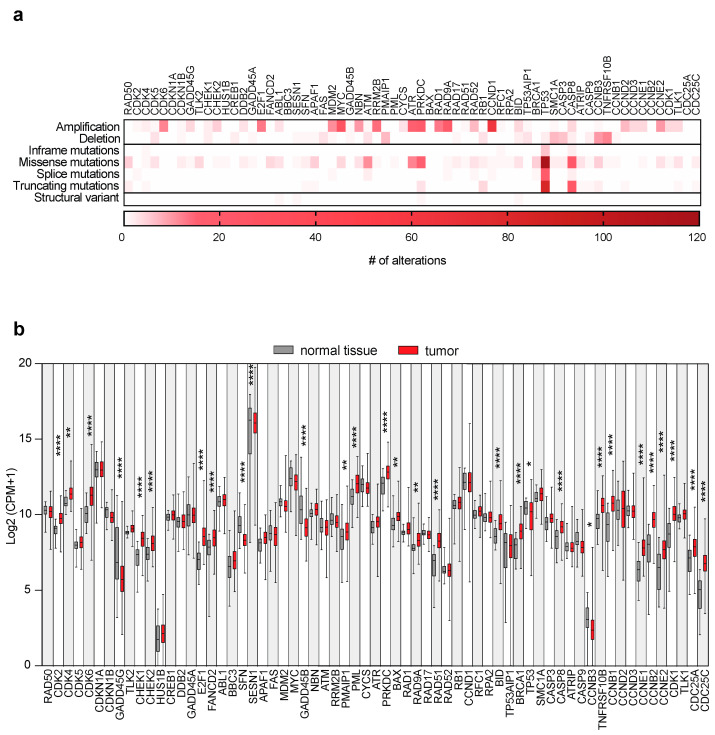
Genomic alterations and expression of DDR genes in human OSCC tumors. (**a**) Heatmap depicting the number of genomic alterations of 65 DDR genes (Supplementary Table S2) in 316 OSCC tumor samples from the TGCA Pan-Cancer Atlas (Supplementary Table S1). (**b**) Box plots depicting RNA-seq analysis of DDR gene expressions in 316 OSCC tumors from the TCGA Pan-Cancer Atlas, and 32 matched normal samples (Supplementary Table S3). One-way ANOVA for multiple comparisons. * *p* < 0.05, ** *p* < 0.01, **** *p* < 0.0001. Groups without statistical significance were unmarked. CPM; counts per million mapped reads.

To evaluate the impact of DDR genes on the biology of OSCC, we selected 316 patients from The Cancer Genome Atlas (TCGA) cohort (Supplementary Table S1) and focused on 65 genes belonging to the DDR Wikipathway collection (Supplementary Table S2). Firstly, we evaluated the gene alteration status of the DDR pathway. Data on gene alteration type and frequency were analyzed using the open source cBioportal. The most common alteration type was copy number amplification, affecting thirty-nine out of sixty-five queried genes. Collectively, twenty genes showed ten or more amplifications. The most affected gene was that coding for the cell cycle controller *CCND1* (65 amplifications), followed by the *MYC* oncogene (28 amplifications), the *PRKDC* gene encoding the DNA-dependent protein kinase catalytic subunit protein (20 amplifications), the *RAD9A* transcription factor (20 amplifications), and the *ATR* kinase gene (16 amplifications) (Figure 1a). Copy number deletions involved twenty-seven out of sixty-five genes, displaying a lower frequency compared to amplifications. Only two genes showed ten or more deletions: *TNFRSF10B* (death receptor 5, 12 deletions) and *CCNB3* (cyclin B3, 10 deletions). Furthermore, extremely low mutation frequency was detected in the OSCC cohort, apart from the *TP53* tumor suppressor gene. In total, 228 mutations accounted for *TP53* with missense mutations (112) as the most common alteration, as already reported [21]. Truncating mutations affected *TP53* (84) and *CASP8* (caspase 8, 24), while splice mutations (except for *TP53*) and structural variants were mostly absent in the analyzed cohort. Overall, the most altered genes were *TP53, CASP8, PRKDC, ATR, MYC*, and *ATM* kinase. To investigate whether the expression levels of the DDR genes were deregulated in OSCC patients, RNA-seq data from 316 tumor samples were compared to the ones from matched normal oral tissues (n = 32; Supplementary Table S3). All the investigated genes were highly expressed, with *SESN1* (coding for the stress-inducible protein sestrin 1) as the most expressed in both OSCC and normal samples, except for *CCNB3* and the *HUS1B* cell cycle checkpoint gene. Among the sixty-five queried genes, thirty showed statistically significant differences between tumor and normal samples (Figure 1b). Six out of thirty genes, namely *CCNB3*, growth arrest and DNA-damage-inducible 45 beta (*GADD45B*) and gamma (*GADD45G*), *SESN1*, *TP53,* and the *SFN* gene coding for the cell cycle checkpoint protein stratifin, were downregulated compared to the normal counterpart. Among the twenty-four upregulated genes, *CDC25C* (coding for tyrosine-protein phosphatase), *CCNE2* (cyclin-E2), *CCNB1* (cyclin-B1), *CHEK1* (coding for serine/threonine-protein kinase), the *E2F1* transcription factor, the *RAD51* DNA repair gene and *CDK6* (cyclin-dependent kinase 6), were the most deregulated compared to normal tissues. Only four out of the twenty-four upregulated genes showed concurrent genomic amplifications (*CDK6, E2F1, PRKDC,* and *CCNE2*). Interestingly, the expression of *TNFRSF10B* and *PMAIP*, a gene involved in the activation of caspases, was upregulated in the analyzed patient’s cohort, despite showing genomic deletions, denoting that this alteration does not represent a mutational hotspot. Altogether, these results suggested the deregulated expression of DDR genes in OSCC patients could not rely on genomic alterations.

### 2.2. DDR Gene Expression Changes Correlate with Grading Parameters of OSCC Patients

**Figure 2:**
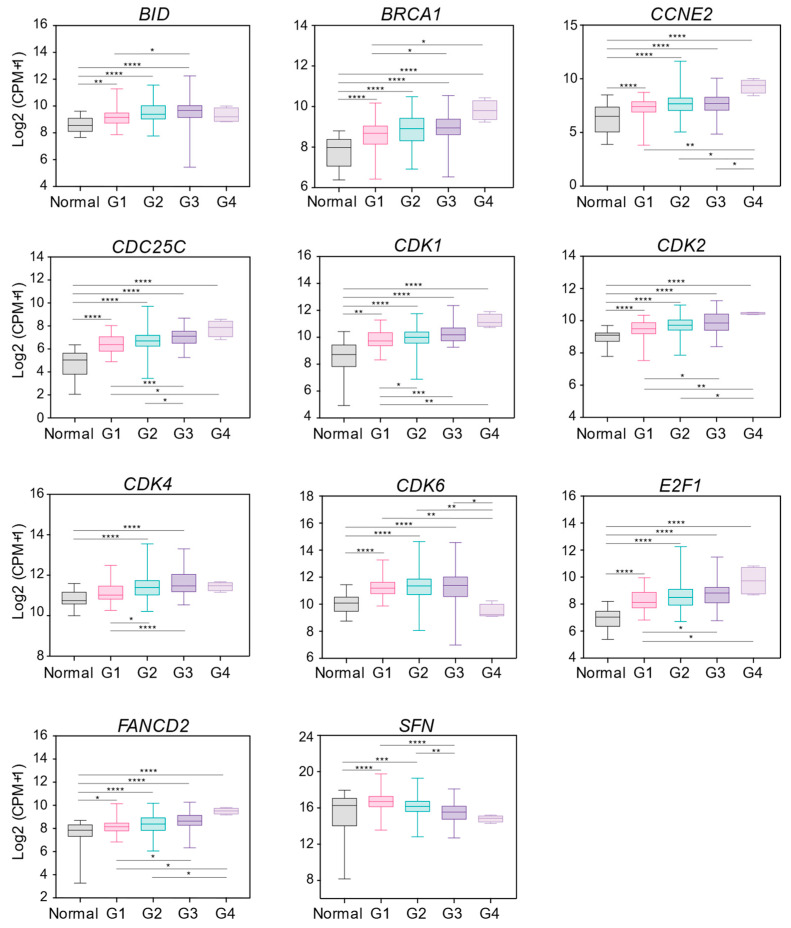
DDR gene expression correlation with clinical parameters in OSCC patients. Box plots showing the correlation between mRNA expression and the tumor grade. G1 (Grade 1) well differentiated (low grade); G2 (Grade 2) moderately differentiated (intermediate grade); G3 (Grade 3) poorly differentiated (high grade); G4 (Grade 4) undifferentiated (high grade). Normal (normal tissue) n = 32, G1 n = 50, G2 n = 189, G3 n = 64, G4 n = 4 (Supplementary Tables S1 and S3). One-way ANOVA for multiple comparisons. * *p* < 0.05, ** *p* < 0.01, *** *p* < 0.001, **** *p* < 0.0001. Groups without statistical significance were unmarked. CPM; counts per million mapped reads.

In OSCC patients, the choice of treatment is mainly based on tumor grade and stage [22]. Thus, we sought to investigate the correlation between the expression of the thirty DDR genes that showed altered mRNA levels compared to normal tissues (Figure 1b) and tumor grade and stage in OSCC patients whose clinical information is available (Supplementary Table S1). As reported in Figure 2, the mRNA expression of eleven genes correlated with the tumor grade. Particularly, ten genes showed a positive correlation between their expression and the individual tumor grade (the higher mRNA expression was associated with the more advanced tumor grade). Conversely, only one gene, the downregulated *SFN*, showed a negative correlation. Among the ten positively correlated genes, seven peaked in grade 4. Surprisingly, the mRNA expression of only one gene, *E2F1*, showed a correlation with tumor stage that did not overlap with the afore-identified genes (Supplementary Figure S1). Together, we individuated ten DDR genes that positively correlated with the clinical grading parameter in OSCC: they were the cell death regulator *BID*, breast cancer type (*BRCA*)1, *CCNE2*, *CDC25C*, cyclin-dependent kinase (*CDK*)1, *CDK2*, *CDK4*, *CDK6*, *E2F1*, and *FANCD2* (coding for Fanconi anemia group D2 protein).

### 2.3. DDR Gene Expression Associates with HPV Infection in OSCC Patients

**Figure 3:**
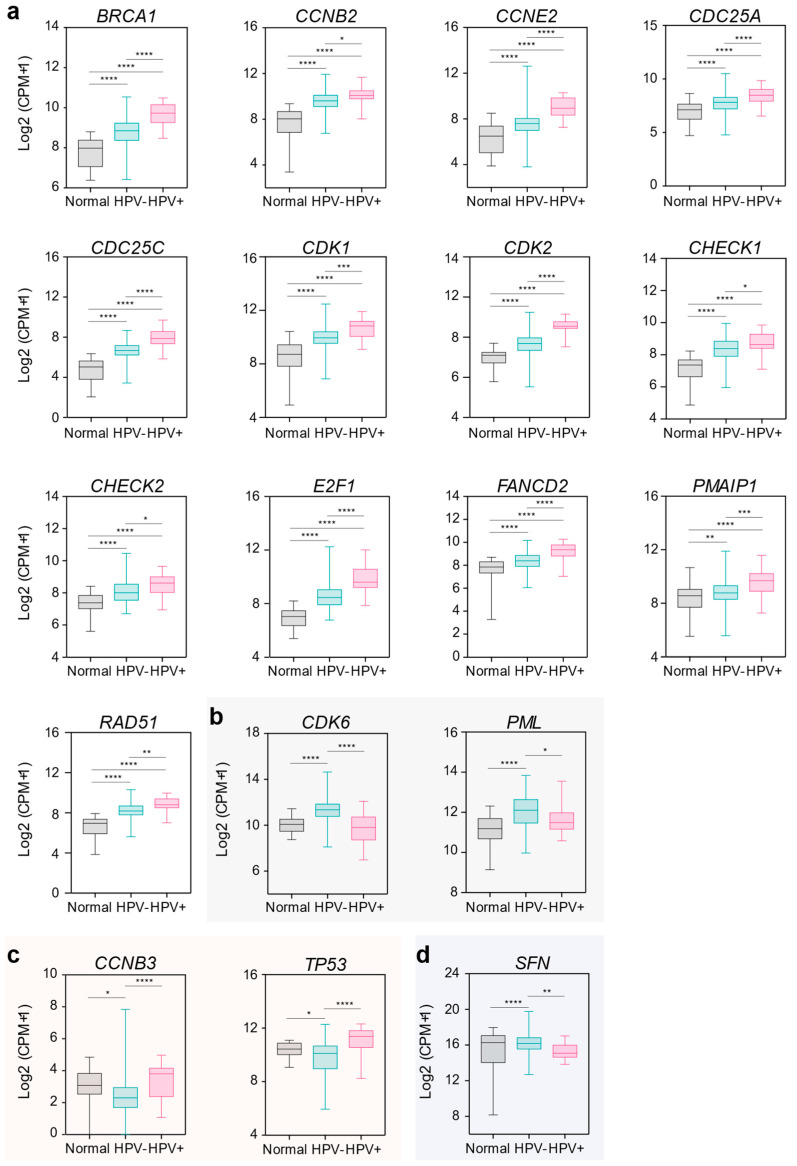
DDR gene expression correlation with HPV infection in OSCC patients. (**a**–**d**) Box plots showing the correlation between mRNA expression and HPV infection. HPV− (negative for HPV infection); HPV+ (positive for HPV infection). Normal (normal tissue) n = 32, HPV− n = 271, HPV+ n = 26 (Supplementary Tables S1 and S3). One-way ANOVA for multiple comparisons. * *p* < 0.05, ** *p* < 0.01, *** *p* < 0.001, **** *p* < 0.0001. Groups without statistical significance were unmarked. CPM; counts per million mapped reads.

Next, we evaluated whether the expression of the thirty DDR genes found to be deregulated in OSCC patients was associated with infection by high-risk HPV known to have a role in OSCC pathogenesis [23]. We identified eighteen genes whose expression was significantly modulated by HPV status in OSCC patients in whom this clinical information was available (Supplementary Table S1). Among them, thirteen genes showed the highest mRNA expression in HPV-positive OSCC patients compared to the HPV-negative ones and healthy controls. They were: *BRCA1*, *CCNB2* (cyclin-B1), *CCNE2*, *CDC25A* (coding for tyrosine protein phosphatase), *CDC25C*, *CDK1*, *CDK2*, *CHEK1*, *CHEK2* (coding for serine/threonine-protein kinase), *E2F1*, *FANCD2*, *PMAIP1*, and *RAD51* (Figure 3a). In contrast, the mRNA levels of *CDK6* and the *PML* transcription factor were upregulated in HPV-negative as compared to HPV-positive OSCCs and normal tissues (Figure 3b). Moreover, we observed that *CCNB3* and *TP53* mRNA levels were lower in HPV-negative OSCCs than in HPV-positive OSCCs and normal tissue (Figure 3c). Noteworthy, the gene expression of the *SFN* gene was lower in HPV-negative OSCCs than in normal tissues, and this downregulation was even stronger in HPV-positive OSCCs (Figure 3d).

### 2.4. BID and CDK2 Expression Impacts on OSCC Patient’s Survival

**Figure 4:**
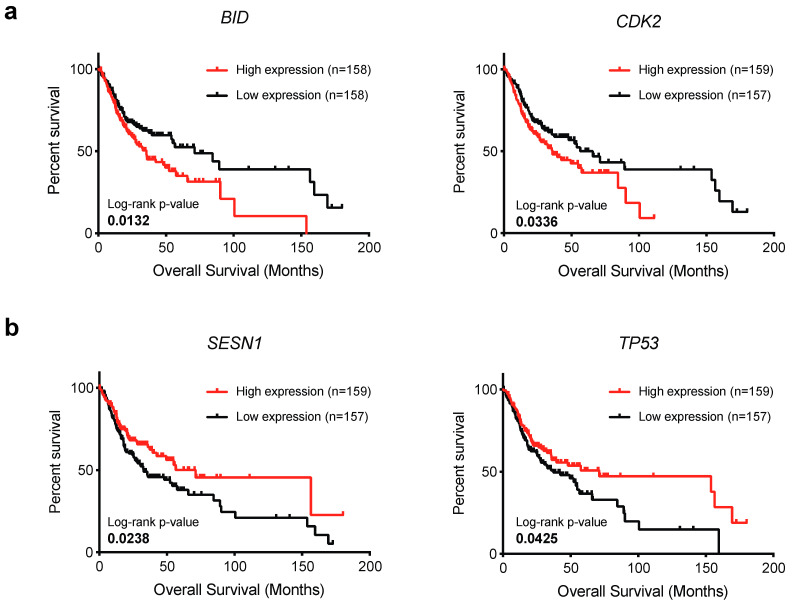
Impact of DDR gene expression on overall survival of OSCC patients. Kaplan–Meier curves depicting the overall survival associated with (**a**) *BID*, *CDK2*, and (**b**) *SESN1* and *TP53* expression of patients affected by OSCC. Significant Log-rank *p*-values are reported in the figure.

To investigate the prognostic value of the thirty deregulated DDR genes, we analyzed the correlation between their mRNA levels and OSCC patients’ overall survival. High mRNA levels of *BID* and *CDK2* significantly correlated with a poor prognosis being associated with shorter overall survival (Figure 4). Conversely, a high expression of *SESN1* and *TP53* was associated with a good prognosis among OSCC patients (Figure 4). These findings were consistent with the downregulation of *SESN1* and *TP53* and the upregulation of *BID* and *CDK2* observed in the OSCC cohort as compared to the normal counterpart (Figure 1). In this context, one should also consider that the expression levels of these genes were significantly correlated with tumor grade (Figure 2) and that *CDK2* was expressed at the highest level in HPV-positive OSCCs. Altogether, these results suggested that *BID* and *CDK2* could usefully monitor OSCC progression and prognosis.

### 2.5. Expression of DDR Genes Affects Survival and Drug Response of OSCC Tumor Cell Lines In Vitro

**Figure 5:**
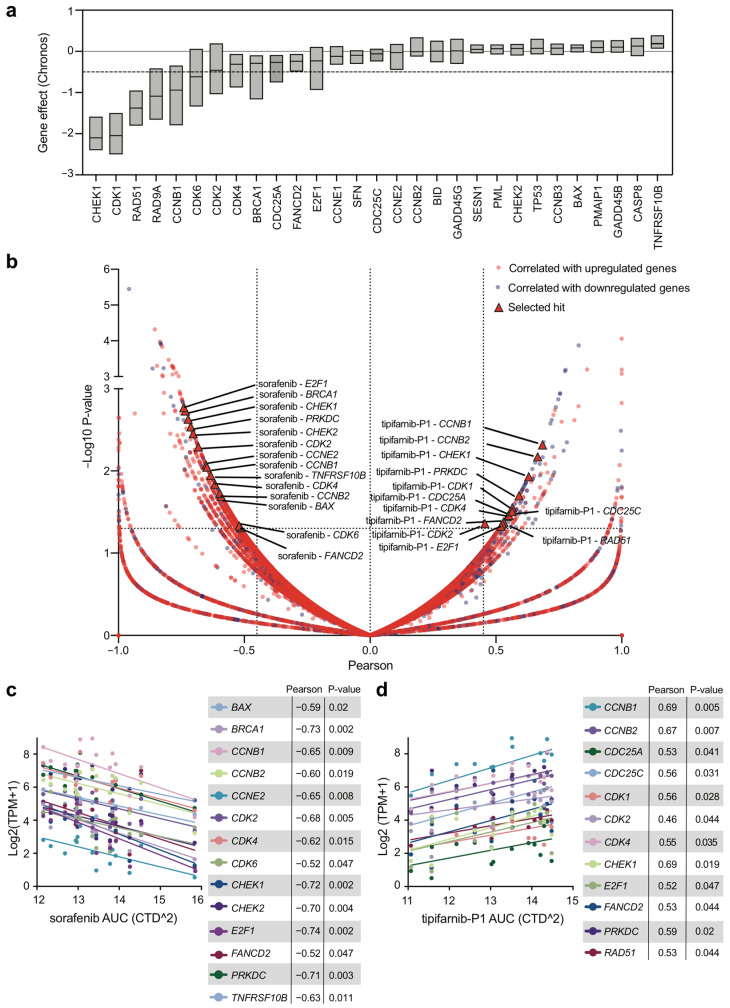
DDR genes perturbation effect and correlation with drug treatment on OSCC tumor cell lines. (**a**) Dependency analysis of DDR genes on OSCC cell lines using CRISPR data from DepMap databases (DepMap22Q2 Public + score, Chronos). The full line settled at 0 indicates no dependency. The dashed line settled at −0.5 indicates survival dependency. (**b**) Volcano plot showing the correlation between DDR gene expressions and drug response in OSCC cell lines from drug sensitivity AUC (CTD ^2). The red dots depict the correlation between upregulated genes and drug response, while the blue dots represent the correlation between downregulated DDR genes and drug response. (**c**,**d**) Pearson correlation analysis between gene expression of the selected DDR genes and drug response to (**c**) sorafenib and (**d**) tipifarnib-P1 from DepMap databases. AUC, area under the curve; TPM, transcript per kilobase million.

The impact of the expression levels of the thirty identified genes on OSCC tumor cell lines was assessed using the DepMap database. Among the 1078 tumor cell lines screened with a genome-wide CRISPR knock-out library, we selected a collection of twenty-five OSCC cell lines (Supplementary Table S4). We then evaluated the effect of the depletion of the DDR genes on cell survival in vitro. As shown in Figure 5a, six genes were identified to be essential for the survival of the OSCC cell lines (*CHEK1*, *CDK1*, *RAD51*, *RAD9A*, *CCNB1*, and *CDK6*) with a dependency score (Chronos) < −0.5. Particularly, CHEK1 and CDK1 depletion showed the highest degree of dependency reaching a score < −2. In this regard, it must be highlighted that both genes were upregulated in OSCC tissues where they correlated with HPV infection and that *CDK1* expression was associated with tumor grade. Moreover, ten genes exhibited a tendency to a vulnerable phenotype with a dependency score < 0 and >−0.5 (*CDK2*, *CDK4*, *BRCA1*, *CDC25A*, *FANCD2*, *E2F1*, *CCNE1*, *SFN*, *CDC25C*, and *CCNE2*). We took advantage of the same DepMap database to investigate whether the expression of the DDR genes was linked to drug response. We analyzed the CTRP v2 portal in which 481 compounds were tested in 860 cancer cell lines, and which reported drug sensitivity connected to the gene expression [24]. A total of fifteen OSCC cell lines were selected (Supplementary Table S5), and the correlation between gene expression of the thirty deregulated DDR genes and the drug response was plotted (Figure 5b). Among the 481 tested compounds, we identified sorafenib, a kinase inhibitor already tested on OSCC cells alone or combined with ionizing radiations [19], as being negatively correlated with the mRNA expression of fourteen DDR genes (*BAX*, *BRCA1*, *CCNB1*, *CCNB2*, *CCNE2*, *CDK2*, *CDK4*, *CDK6*, *CHEK1*, *CHEK2*, *E2F1*, *FANCD2*, *PRKDC*, and *TNRFSF10B*) (Figure 5b). The drug treatment response with sorafenib resulted in being more efficient (low AUC values) in cell lines with higher expression of the genes (Figure 5c).

**Figure 6:**
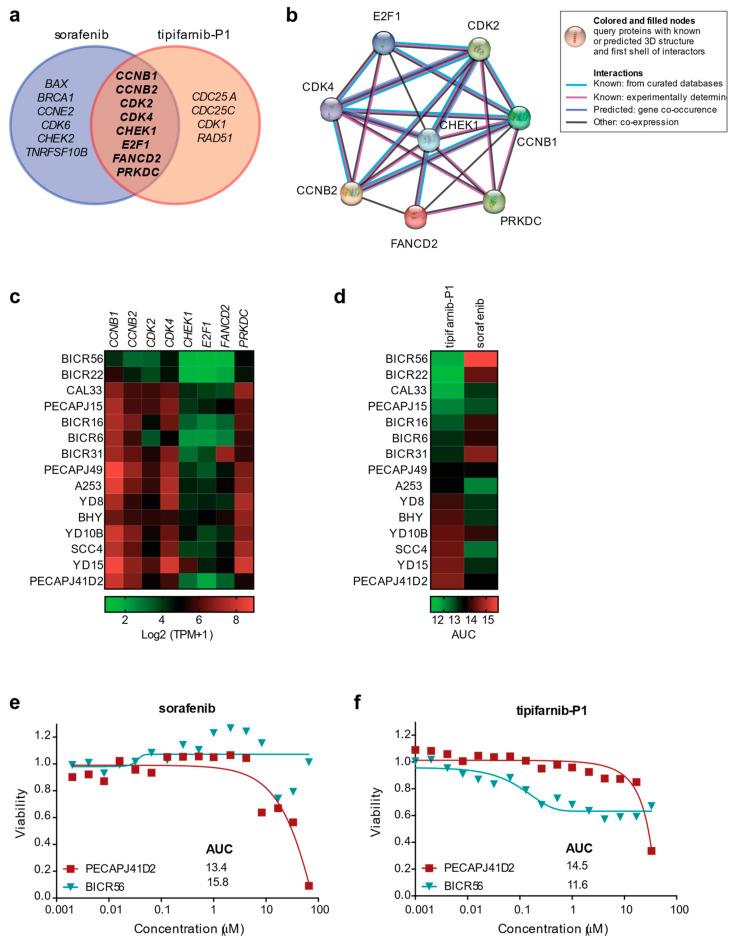
Gene signature of drug response in OSCC cell lines. (**a**) Venn diagram showing the integration of the response signature to sorafenib and tipifarnib-P1. (**b**) STRING protein–protein interaction networks functional enrichment analysis of eight DDR genes correlated to drug response identified in (**a**). (**c**) Heatmap depicting mRNA expression of eight DDR genes in OSCC cell lines. TPM, transcript per kilobase million. (**d**) Heatmap showing drug response to tipifarnib-P1 and sorafenib in OSCC cell lines. AUC, area under the curve. (**e**,**f**) Drug response curve to (**e**) sorafenib and (**f**) tipifarnib-P1 in two selected OSCC cell lines, expressing low (BICR56) and high (PECAPJ41D2) mRNA levels of DDR genes retrieved from drug sensitivity AUC (CTD ^2).

By employing the same approach, we found that OSCC cell responsiveness to tipifarnib-P1, a farnesyltransferase inhibitor exploited for the “pathogenetic” therapy of HRAS mutant-head and neck SCC [20], correlated with the expression of twelve genes (*CCNB1*, *CCNB2*, *CDC25A*, *CDC25C*, *CDK1*, *CDK2*, *CDK4*, *CHEK1*, *E2F1*, *FANCD2*, *PRKDC*, and *RAD51*). Specifically, the more expressed the genes, the less effective (high AUC values) the treatment with tipifarnib-P1 (Figure 5d). Finally, by integrating the fourteen and twelve genes correlated with the response to sorafenib or tipifarnib-P1, respectively, we identified eight DDR genes (*CCNB1*, *CCNB2*, *CDK2*, *CDK4*, *CHEK1*, *E2F1*, *FANCD2*, and *PRKDC*) (Figure 6a,b), the upregulation of whose mRNAs could predict the success of OSCC patients’ treatment (Figure 6c–f).

Altogether, these data constitute a gene signature to be employed to select OSCC patients for the appropriate therapy.

## 3. Discussion

OSCC is a highly aggressive and metastasizing cancer [25,26]. Therapeutic treatment involves surgical exeresis, whenever this is possible, and/or the use of DNA damage inducers such as radiotherapy or cytotoxic chemotherapy [7]. The choice of the right therapeutic strategy is strongly influenced by the histologic grade of the tumor, its localization, and the extension of the lesion, along with the presence of OSCC developmental or progression factors such as HPV infection [8]. However, the differentiation-based histopathologic grading system has still low prognostic value in OSCC [27], and the contribution of HPV infection to OSCC needs to be further clarified [6]. As a result, the rate of resistance to therapy and the development of relapses are remarkable [28]. For these reasons novel prognostic biomarkers and therapeutic approaches are urgently needed.

Although comprehensive genomic analyses of gene expression, copy number alteration, and mutation have been recently performed in OSCC patients [29], no prognostic markers or therapeutic targets have been identified. Therefore, we exploited TCGA to retrieve data from the OSCC patient cohort and further investigated the implication of DDR genes in OSCC tumorigenesis.

Our results indicate that copy number amplification is the most frequent alteration in the analyzed OSCC cohort, affecting 60% of the investigated DDR genes. Conversely, copy number deletions affect 40% of the genes with a lower frequency. By comparing the DDR gene expression of the OSCC cohort with the normal oral tissue counterpart, we have identified thirty differentially expressed genes. Integration of genomic alteration and expression data highlights that the dysregulated expression of the DDR genes in the OSCC cohort could not rely only on the mutational profile, suggesting epigenetic and transcriptional regulation involvement. Indeed, only six genes (*CCNB3*, *CCNE2*, *CDK6*, *E2F1*, *PRKDC,* and *TP53*) have shown agreement between genomic alteration and gene expression.

Our analysis has identified the tumor suppressor *TP53* gene as the most altered gene. This finding is in accordance with recent evidence from genomic sequencing analyses which have reported a high incidence (65–85%) of *TP53* mutations in OSCC [21,30]. Mutations are predominately localized in the DNA-binding domain of p53: this blocks p53 ability to transactivate downstream target genes [21,30], thereby impairing p53 tumor suppressor function and leading to OSCC development and progression [21,29,31,32]. Consistently, we have also observed that high *TP53* expression correlates with a good prognosis in OSCC patients.

In the present study, we have also found that *CASP8*, a key controller gene of apoptosis, is frequently mutated in OSCC tissues. Interestingly, a new molecular subtype of OSCC, characterized by several mutations and few copy number alterations of *CASP8* has been recently described [32], highlighting its role in carcinogenesis when deregulated. Moreover, an increased frequency of *CASP8* mutations has been reported in oral tumor tissues as compared to preneoplastic lesions such as leukoplakia [33].

Among the most amplified gene in OSCC, we have identified *CCND1*. This finding is in agreement with the fact that cell-cycle alterations are common features of cancer cells, and that focal amplicon on chromosome 11, including *CCND1*, have been reported in OSCC [34]. Nevertheless, we have found no altered *CCND1* mRNA levels in OSCCs as compared to normal oral tissue, suggesting the inactivation of the amplified allele and/or the synthesis of noncoding regulatory RNAs derived from the DNA amplicon.

Results from our analysis also indicate that, among the thirty genes which are differentially expressed in OSCCs, seven of them (namely *BRCA1*, *CCNE2*, *CDC25C*, *CDK1*, *CDK2*, *E2F1,* and *FANCD2*) positively correlate with both tumor grade and HPV infection. Among the differentially expressed DDR genes, two significantly correlate with a poor prognosis: *BID* and *CDK2*. The latter, a cyclin-dependent kinase, participates in cell cycle progression. Its binding to Cyclin E is essential for G1 transition, while the subsequent binding to Cyclin A is involved in S phase progression [35]. Previous studies have reported that increased expression of *CDK2* is a critical factor for the progression of oral cancer and can be used as a predictive marker for poor prognosis [36]. In our study, in addition to confirming that *CDK2* is a marker of poor prognosis, we have found that this gene harbors genomic amplifications and splice mutation and that its expression: (i) is upregulated in OSCC patients; (ii) correlates with cancer grade; and (iii) is associated to HPV infection. Altogether, these data suggest that DDR gene alterations, both at the genomic and transcriptomic levels, could be taken into consideration in the development of a molecular diagnostic procedure.

Recently, the efficacy of inhibitors of the molecular mechanisms leading to OSCC onset and progression has also been tested. Among them, sorafenib and tipifarnib-P1 possess promising activities against OSCC [19,20].

Sorafenib is a multikinase inhibitor that functionally hampers both the Nuclear Factor kappa B transcription factor and the repair of DNA damage in head and neck SCC cells. In doing so, sorafenib enhances the anti-OSCC activity of ionizing radiations [19,37] and, when combined with chemotherapeutics, inhibits the growth, migration, and invasion of OSCC cells [38]. To date, seven clinical trials are testing sorafenib combined or not with radiation, chemotherapy, or cetuximab in patients affected by head and neck SCC. Five of the trials (three Phase I and two Phase II) have been completed, one (Phase I) is withdrawn, and another one (Phase II) is active but not recruiting. Of note, no genetic or transcriptomic characteristics have been added as inclusion criteria.

With regard to tipifarnib-P1, it binds and potently inhibits the farnesyltransferase enzyme which mediates RAS activation [39]. For this reason, tipifarnib-P1 has become a pan-RAS targeted therapy, and a specific *HRAS* mutant treatment, being HRAS, but not KRAS or NRAS, exclusively dependent on farnesylation [40]. Currently, three clinical trials are evaluating tipifarnib-P1 efficacy in head and neck SCC: one (Phase II) has been completed, another one (Phase I/II) is recruiting, and the third one (Phase II) is active but not recruiting. *HRAS* mutation or overexpression is an inclusion criterion for enrollment.

Definitely, the development of personalized, molecularly targeted therapies is needed to prolong the survival and ameliorate the quality of life of OSCC patients. Aimed at providing information employable to design tailored anti-OSCC strategies possibly less toxic and more effective than the conventional ones, we have taken advantage of a large drug screening to correlate DDR gene expression with drug response. We report here, for the first time, a signature based on the expression of eight genes (namely *CCNB1*, *CCNB2*, *CDK2*, *CDK4*, *CHEK1*, *E2F1*, *FANCD2*, and *PRKDC)* which can predict the response of OSCC cells to the novel anti-OSCC drugs sorafenib and tipifarnib-P1. In particular, we have observed that the expression of the eight abovementioned genes negatively correlates with the sensitivity of OSCC cells to sorafenib, while positively associating with the responsiveness of OSCC cells to tipifarnib-P1.

Taken together, our data demonstrate that the dysregulated expression of the DDR pathway in OSCC could offer a potentially useful prognostic signature, predictive of drug response. Indeed, the use of markers for factors involved in OSCC pathogenesis could help clinicians formulate more effective treatment protocols than those now available for OSCC patients. 

Notably, the use of publicly available genomic data on a large cohort of OSCC patients from TCGA has enabled the examination of a clinically relevant question in a large dataset that has been collected worldwide and over time. Nevertheless, our data should be confirmed with in-depth in vitro and in vivo analyses.

## 4. Materials and Methods

### 4.1. Head and Neck Oral Squamous Cell Carcinoma Data

TCGA sample IDs and clinical information for head and neck SCC and matched normal tissues were downloaded from the Xena database [41] (accessed on 16 September 2022). Only entries with both RNA count data and clinical information were included. A selection for the anatomic site of the neoplasm was made to define the OSCC patient subset used for the subsequent analyses. The following anatomic sites were included: base of the tongue, oral tongue, oral cavity, buccal mucosa, hard palate, and floor of the mouth. No overlapping samples were considered. Slovin’s Formula was applied to assess the minimum sample size of OSCC samples over the head and neck SCC TCGA dataset.

### 4.2. DNA Damage Response Gene List

The DDR gene list was downloaded from the GSEA website at the following link: http://www.gsea-msigdb.org/gsea/msigdb/cards/WP_DNA_DAMAGE_RESPONSE.html (accessed on 16 September 2022). The 65 queried coding genes, listed in Supplementary Table S1, belong to the WikiPathways collection DNA_DAMAGE_RESPONSE.

### 4.3. Alteration Frequency Analysis

DNA alteration frequency data of the selected OSCC patient cohort was downloaded from cBioPortal for cancer genomics (https://www.cbioportal.org/) (accessed on 16 September 2022). Amplification, deletion, inframe mutations, missense mutations, splice mutations, truncating mutations, and structural variants from whole genome sequencing data were considered. Data were analyzed and plotted as a heatmap using GraphPad Prism 8.0 (San Diego, CA, USA).

### 4.4. Gene Expression Analysis

The expression of DDR genes in the OSCC patient cohort and matched normal samples were downloaded from the Xena database [41] as per developer instructions (accessed on 16 September 2022). The recovered RNA-seq data are expressed as Log2 (CPM + 1). Statistical significance has been calculated using one-way ANOVA for multiple comparisons, and only comparisons with a *p*-value ≤ 0.05 have been considered significant. Data were analyzed and plotted as box plots using GraphPad Prism 8.0.

### 4.5. Survival Analysis

Overall survival data from OSCC patients were downloaded from cBioportal for Cancer Genomics (accessed on 7 November 2022). Association analysis between DDR gene expression and overall survival data was performed using GraphPad Prism 8.0. *p*-values are given using the log-rank test, and data are dichotomized into high or low expression of DDR genes based on the median value and reported as survival probability. Data are plotted and represented as a Kaplan–Meier picture.

### 4.6. Dependency Analysis

Data from CRISPR genome screening on 25 OSCC cell lines (Supplementary Table S4) were downloaded from DepMap (https://depmap.org/portal/; DepMap 22Q4 Public + score, Chronos) (accessed on 5 December 2022). Perturbation gene effects are reported as Chronos. Data were analyzed and plotted as floating bar plots using GraphPad Prism 8.0.

### 4.7. Gene Expression and Drug Sensitivity Analysis

Correlations between DDR gene expression and drug sensitivity in 15 OSCC cell lines (Supplementary Table S5) have been retrieved from DepMap using the 22Q2 public dataset for gene expression and Drug Sensitivity AUC (CTD ^2) dataset for drug response data. (https://depmap.org/portal/) (accessed on 5 December 2022). Only correlations with a Pearson coefficient < −0.45 and >0.45 and *p*-values ≤ 0.05 have been considered. Data were analyzed and plotted as a volcano plot using GraphPad Prism 8.0.

### 4.8. Statistical Analysis

*p*-values have been calculated with GraphPad Prism 8.0 using one-way ANOVA for multiple comparisons. *p*-values ≤ 0.05 have been considered statistically significant and are reported within the Figures, * *p* < 0.05, ** *p* < 0.01, *** *p* < 0.001, **** *p* < 0.0001. Groups without statistical significance were unmarked.

## Data Availability

The data that support the findings of this study are openly available and were downloaded from the cBioportal database (https://www.cbioportal.org) (accessed on 16 September 2022), the UALCAN database (http://ualcan.path.uab.edu) (accessed on 16 September 2022), and the DepMap database (https://depmap.org/portal/) (accessed on 5 December 2022).

## Supplementary Materials

The following supporting information can be downloaded at: https://www.mdpi.com/article/10.3390/ijms24032673/s1.
