# Supplementary material for: DNA Damage Response Gene Signature as Potential Treatment Markers for Oral Squamous Cell Carcinoma
Source: Int J Mol Sci. 2023 Jan 31;24(3):2673. doi: 10.3390/ijms24032673 (PMC9916929; doi:10.3390/ijms24032673)
Supplement: Supplementary file 1 [file ijms-24-02673-s001.zip › Supplementary Figures.docx]

**Supplementary Figures**


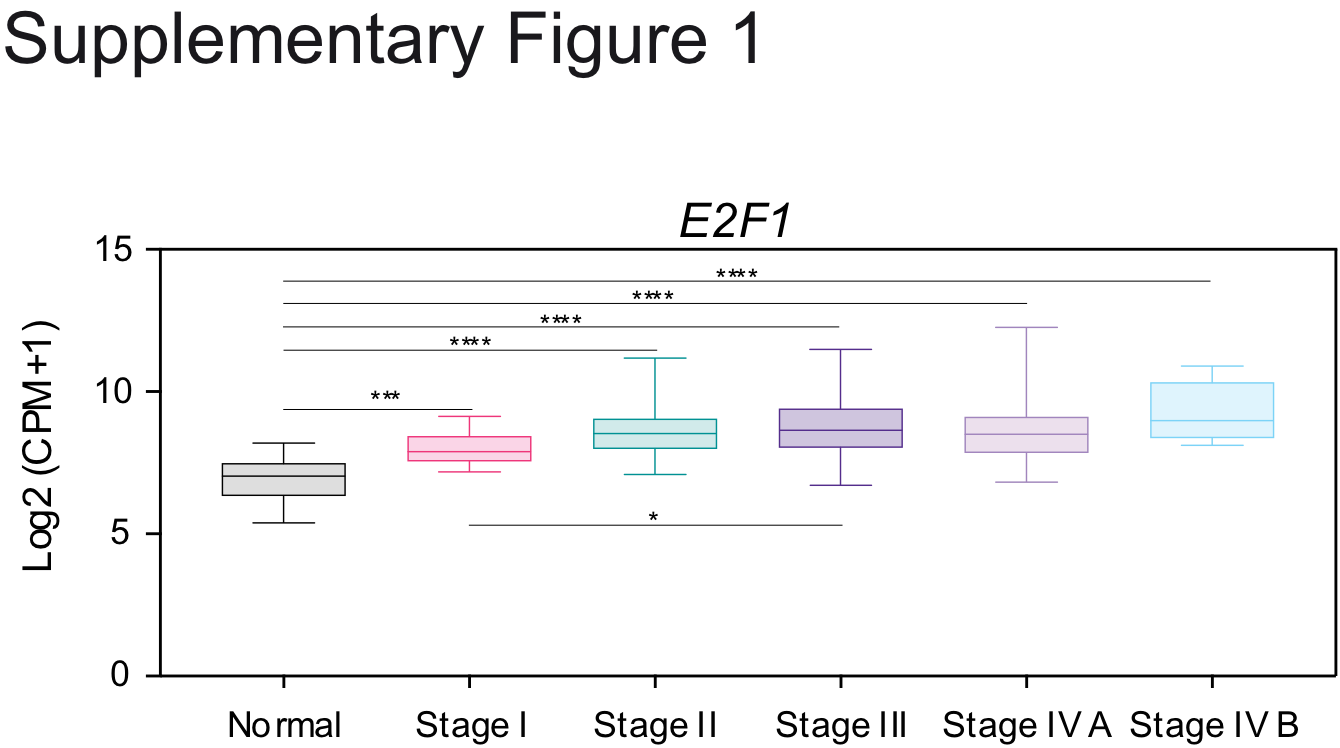


**Supplementary Figure 1.** Gene expression correlation with staging parameters in OSCC patients. Box plots showing correlation between *E2F1* mRNA expression and the tumor stages. Normal (normal tissue) n=32, Stage I n=18, Stage II n=54, Stage III n=60, Stage IV A n=149, Stage IV B n=5 (Supplementary Tables 1 and 3). One-way ANOVA for multiple comparisons. *p < 0.05, ***p < 0.001, ****p < 0.0001. Groups without statistical significance were unmarked. CPM, counts per million mapped reads.
