# Supplementary figures and images for: DNA Damage Response Gene Signature as Potential Treatment Markers for Oral Squamous Cell Carcinoma
Source: Int J Mol Sci. 2023 Jan 31;24(3):2673. doi: 10.3390/ijms24032673 (PMC9916929; doi:10.3390/ijms24032673)

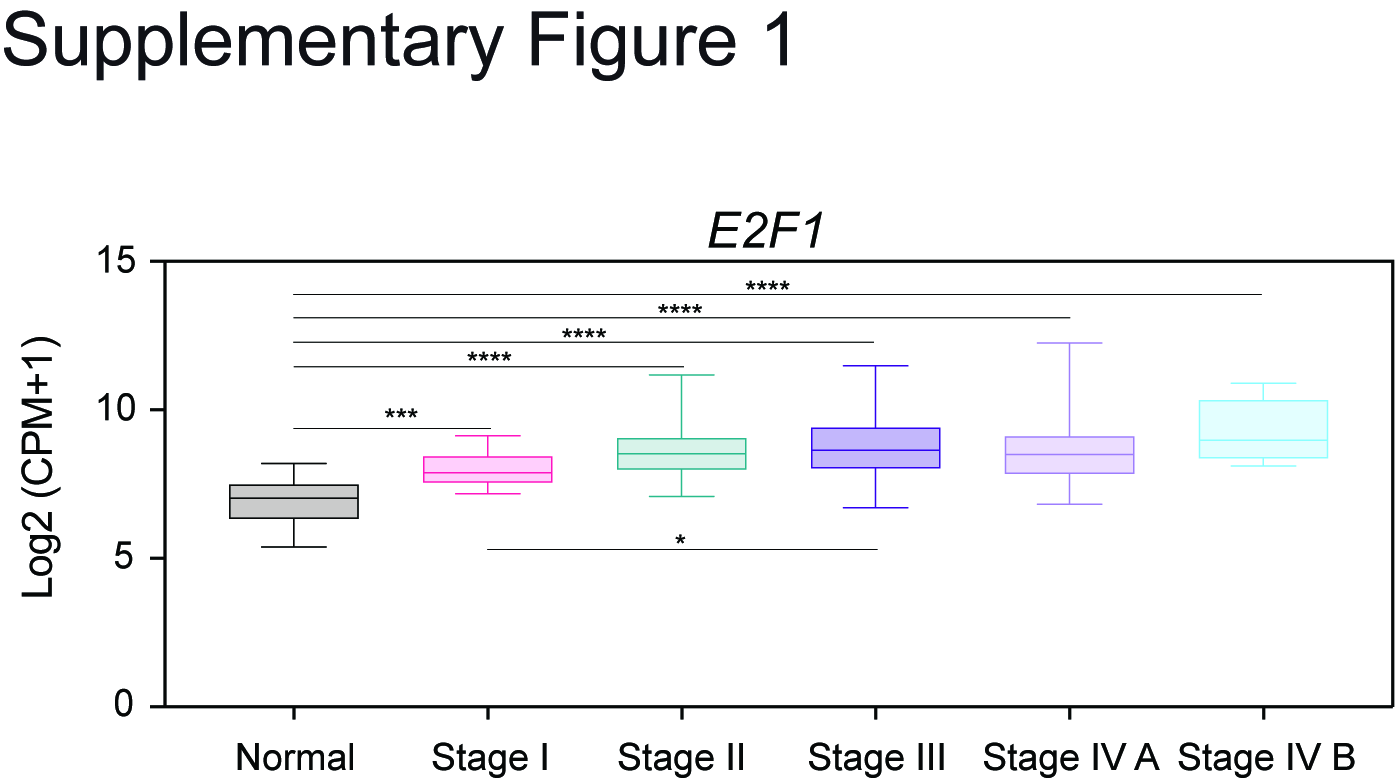

Supplement: Supplementary file 1 [file ijms-24-02673-s001.zip › Figure S1.tif]
